# The Effect of Geometric and Material Nonlinearities on the Development of Membrane Resistance in Reinforced Concrete Flat Slab–Column Buildings

**DOI:** 10.3390/ma18174053

**Published:** 2025-08-29

**Authors:** Sylwester Walach, Seweryn Kokot, Juliusz Kus

**Affiliations:** Faculty of Civil Engineering and Architecture, Opole University of Technology, 45-758 Opole, Poland; s.kokot@po.edu.pl (S.K.); j.kus@po.edu.pl (J.K.)

**Keywords:** reinforced concrete, flat-slab column structure, progressive collapse, pushdown, finite element analysis, column removal

## Abstract

This article presents a numerical study of the influence of applied nonlinearities on the response of a flat slab–column structure under progressive collapse conditions. A key aspect of the work is the extension of nonlinear static analysis by considering cases of material nonlinearity combined with both linear and nonlinear geometry, using a corotational formulation and a damage-based elasto-plastic concrete model. A multi-layer shell element implemented in the OpenSees platform is used to distinguish between the strength characteristics of the concrete and reinforcement, with particular attention given to the modeling of the slab–column connection in nonlinear analyzes involving both shell and beam elements. The applied vertical pushover analysis enabled the derivation of load–displacement curves and the identification of the sequence in which plastic hinges can be formed. The development of membrane action resistance, expressed through the formation of compressive and tensile rings, is observed numerically when both material and geometric nonlinearities are simultaneously considered. Moreover, the transition from compressive membrane action to tensile membrane action occurs once the deflections reach the value equal to the effective depth of the slab. This insight may serve as an important guideline for the development of future revisions to design standards related to progressive collapse.

## 1. Introduction

Progressive collapse in buildings can be initiated by the local failure of a primary load-bearing element such as a column, beam, wall, or slab, and may subsequently propagate through the structural system, ultimately resulting in global collapse. The phenomenon is recognized in major design codes and guidelines, including the European Eurocodes [1], the British Standard BS 6399 [2], and U.S. provisions published by the Department of Defense (DoD) [3,4], the General Services Administration (GSA) [5,6], and the National Institute of Standards and Technology (NIST) [7,8]. These documents identify exceptional loading scenarios as potential triggers of collapse, such as gas explosions (e.g., the Ronan Point partial collapse [9,10]), terrorist attacks (e.g., the World Trade Center towers [11,12] and the Murrah Federal Office Building [13,14,15,16]), and extreme environmental events (e.g., the Tacoma Narrows Bridge collapse [17]). Additional examples are provided in [18].

**Figure 1:**
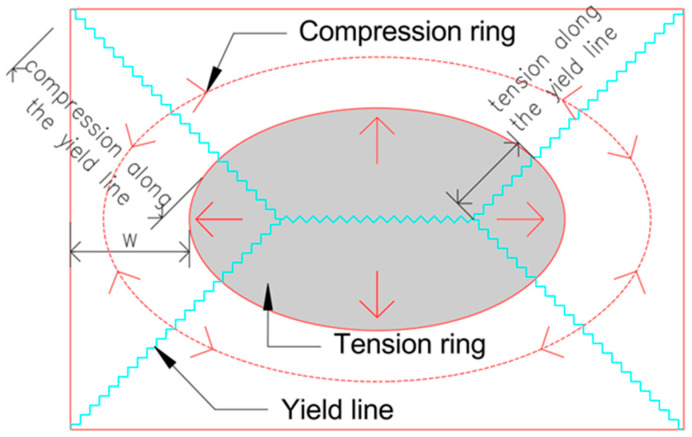
Membrane force development in flat slab–column structures at large deformations.

Progressive collapse has been widely investigated in reinforced concrete frames [19,20,21,22,23,24,25,26,27] and flat slab–column systems [28,29,30,31]. Valuable, though limited, insights have been obtained from full-scale experimental programs [32,33,34,35] and tests on subassemblies [36,37,38,39]. A fundamental research gap persists: the numerical capture of compression membrane action (CMA) and tension membrane action (TMA) in slabs is only possible when both geometric and material nonlinearities are incorporated simultaneously. If either nonlinearity is introduced in isolation, these resistance mechanisms do not develop. Figure 1 provides the reference schematic used throughout this study, depicting the circumferential compressive ring and the interior tensile membrane forces. The width of the compressive ring (denoted W in Figure 1) decreases progressively with increasing deflection until failure, as stresses concentrate near the slab perimeter to equilibrate the central tensile forces.

The finite element method (FEM) is the most widely used numerical tool for progressive collapse analysis. Both two- and three-dimensional models are applied, typically classified into micro-modeling approaches [40,41,42,43] and macro-modeling strategies [44,45,46,47]. Computational analyzes are generally grouped as linear or nonlinear and as static or dynamic. Among these, nonlinear static pushdown analysis—conceptually analogous to pushover analysis in seismic engineering—is commonly adopted to evaluate residual load-carrying capacity following column removal [48,49,50,51,52,53,54].

Collapse resistance depends strongly on the activation of specific mechanisms. Following the loss of a column, structures may undergo distinct stages of redistribution [55], and the increase in effective span can amplify negative bending moments by up to fourfold above the removed support [56]. In frame systems, Vierendeel action [57,58], compressive arch action (CAA), and catenary tension action (CTA) are predominant [39,59,60,61,62]. In flat slab–column systems, yield-line mechanisms may precede the development of CMA and TMA [37,63,64,65]. As noted earlier and illustrated in Figure 1, the formation of the circumferential compressive ring governs subsequent failure modes, including corner crushing, mid-span reinforcement rupture, or rupture at plastic-hinge intersections [39,66,67,68]. Boundary conditions and reinforcement ratios further influence slab capacity; for example, lateral confinement by adjacent spans can increase flexural resistance by up to 17% [29].

To reduce computational cost, some studies have neglected slab stiffness altogether or represented it approximately using equivalent stiffness models [13,48,69]. This can underestimate progressive collapse resistance by up to a factor of 1.5 compared with models that explicitly include slab behavior [70]. Furthermore, as demonstrated in [71], membrane action in slab–beam systems may inhibit the simultaneous yielding of all reinforcing bars in a beam cross-section, undermining assumptions commonly adopted in simplified approaches.

Accordingly, the objectives of this study are to (1) examine the influence of geometric nonlinearity while assuming a linear elastic concrete model; (2) assess the effect of a damage-based elasto-plastic concrete model under linear geometry; and (3) evaluate the combined effect of geometric and material nonlinearities using pushdown analysis.

The original contributions of this study are as follows:

1. Demonstrating that CMA and TMA mechanisms do not arise when only geometric or only material nonlinearity is included;
2. Establishing that their numerical occurrence in slab-type structures is feasible only when both geometric and material nonlinearities are incorporated simultaneously.

The paper is organized as follows. Section 2 describes the numerical model of the analyzed flat slab–column structure. Section 3 examines results obtained when only geometric nonlinearity is considered and compares them with linear analysis. Section 4 contrasts pushdown curves from a fully nonlinear model with those from a model incorporating material nonlinearity but assuming linear geometry, highlighting the evolution of membrane action as a function of deflection. Finally, Section 5 and Section 6 summarize the findings, draw conclusions, and outline directions for future research. All numerical models and simulations are performed using OpenSees [72], with the graphical interface STKO [73]. All shell elements, constitutive material models, and structural definitions discussed in the following sections refer specifically to implementations available within this framework.

## 2. Computational Model of the Analyzed Structure

To better understand the influence of large displacements and the formation of CMA and TMA, a reinforced concrete slab with a thickness of 0.20 m and dimensions of 9.0 m × 6.0 m is analyzed. The slab represents a virtually isolated portion of a larger flat slab–column structural system.

**Figure 2:**
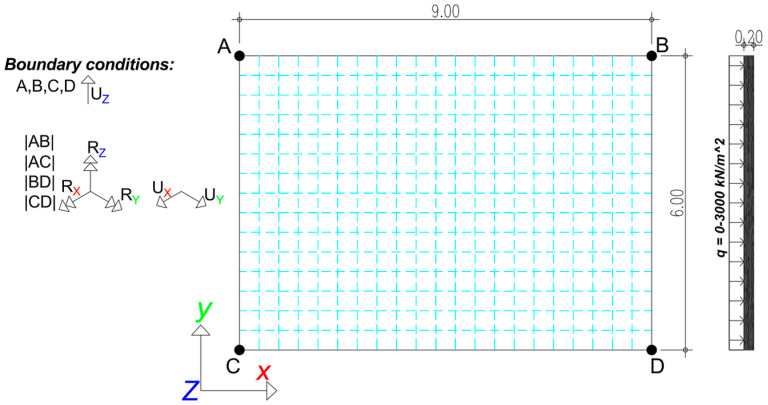
Model of the slab under elastic concrete material behavior and nonlinear geometry conditions.

In the first example (Section 3), the slab (Figure 2) is analyzed under the assumption that vertical displacements (UZ) are restrained at four discrete corner points (A, B, C, D), simulating the presence of columns implicitly. Along the slab’s linear edges, the rotational degrees of freedom (RX, RY, RZ) and in-plane translations (UX, UY) are restrained, representing a simplified model of a central span with lateral confinement provided by adjacent structural elements (i.e., laterally restrained slabs). The slab is subjected to incrementally applied uniform vertical surface loading, ranging from 0 to 3000 kN/m^2^.

**Figure 3:**
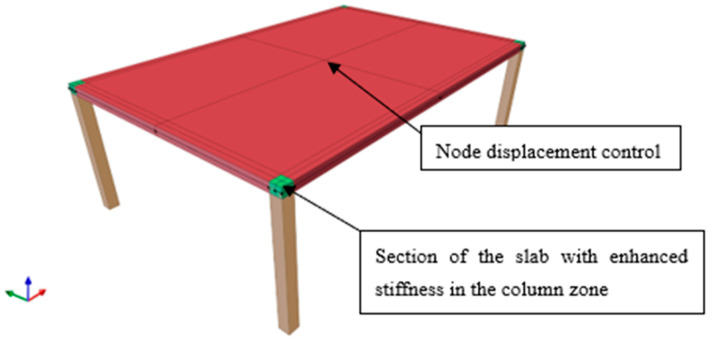
Model of the slab used in the nonlinear pushdown analysis (dimension as in Figure 2).

In the second example (Section 4), the columns are modeled as elastic frame elements with cross-sectional dimensions of 0.25 m × 0.25 m and a height of 3.00 m. The columns are rigidly connected to the slab and fully fixed at their bases (Figure 3). All other boundary conditions remained unchanged. Using a displacement-controlled analysis method [74,75], the vertical displacement at the center of the slab span is incrementally increased. The maximum load is identified at the point beyond which the numerical solution failed to converge.

It is important to note that in nonlinear analyzes, especially in the case of slabs, stress concentrations above the columns may lead to premature (non-physical) failure, convergence issues, and a high sensitivity of the results to the mesh density. To mitigate these effects, concentrated loads and direct connections to single nodes should be avoided.

This study proposes a column-to-slab connection modeling strategy, which, in the authors’ view, is insufficiently addressed in the existing macro-modeling literature. In the example under consideration, the columns are offset from the slab edges so that their cross-sectional area remains fully within the slab boundaries. Additionally, to reflect the significantly higher stiffness of the joint region, which in real structures is ensured by intersecting reinforcement, this zone is modeled as linear elastic (highlighted in green in Figure 3) with a stiffness 100 times greater than that of the surrounding material (E = 3.0 × 10^10^ kN/m^2^). This modeling approach ensures that slab failure initiates at the slab–column interface rather than along the column centerline, reproduces a realistic post-failure condition in which part of the column remains beneath and/or above the slab following a localized damage event, such as an explosion, and accurately represents the fixed constraints at the slab–column connection.

All shell elements are modeled using the four-node quadrilateral ASDShellQ4 element [76], which employs 2 × 2 Gauss integration points. ASDShellQ4 is a general-purpose element suitable for both thick and thin slab applications. It can be used to model flat as well as warped geometries and exhibits the following characteristics: (1) The membrane behavior is improved by the AGQ6-I formulation [77], making the element practically insensitive to geometry distortion compared with standard isoparametric elements. (2) The Hughes–Brezzi formulation [78] is applied to the drilling DOF using a 1-point quadrature plus stabilization, taking extra care to prevent membrane locking. (3) To prevent the well-known transverse shear locking behavior of thick plate elements, the MITC4 formulation is applied to the plate bending section [79,80]. (5) Geometric nonlinearity in the ASDShellQ4 element is implemented based on the work presented in [81,82], using an element corotational formulation. This approach allows for the consideration of large displacements and rotations, which significantly influence the distribution of internal forces and the overall structural response. Due to the use of nonlinear kinematics, the internal forces (i.e., equilibrium equations) at each step of the analysis are computed with respect to the deformed geometry rather than the original, undeformed configuration.

**Figure 4:**
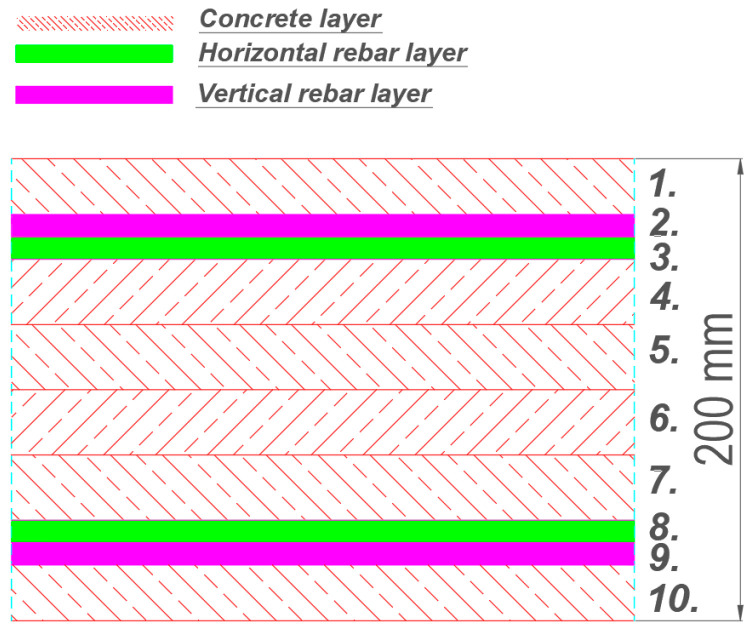
A typical multi-layered shell element for modeling of reinforced concrete slabs.

The reinforced concrete slab cross-section is modeled using a multi-layer shell element implemented in OpenSees, based on the methodology described in [83,84,85]. This formulation follows the principles of composite material mechanics and consists of multiple layers with varying thicknesses and material properties (e.g., concrete layers and/or reinforcing bar layers). An example of a reinforced concrete slab cross-section with a total thickness of 200 mm is shown in Figure 4.

**Figure 5:**
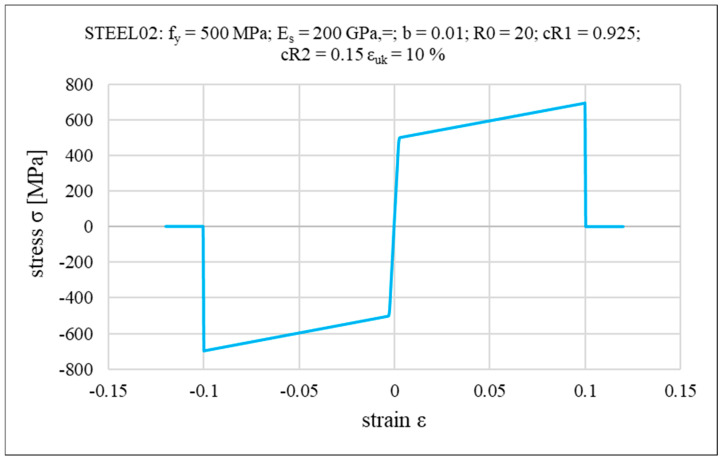
Backbone of the stress–strain relationships of steel.

Layers 1 and 10 represent the concrete cover (1 and 10 = 30 mm), while layers 4 through 7 correspond to the core of the slab (35 mm each). The total thickness of the concrete layers equals the full slab height, h = 200 mm. Layers 2, 3 and 8, 9 represent smeared longitudinal and transverse reinforcement in the top and bottom reinforcement zones. After computing the axial strain and curvature of the mid-layer, the assumption of plane sections is used to determine strains in the remaining layers. It is assumed that the stress across the thickness of each layer is uniform and corresponds to the stress at its mid-surface. Therefore, if the slab is divided into a sufficient number of layers, the multi-layer shell element can accurately capture the through-thickness stress distribution [86]. In this study, the slab is divided into 20 concrete layers (each 10 mm thick) and 4 reinforcement layers. The thickness of a single reinforcement layer is calculated according to the following equation:

(1) $$t_{layer}=\frac{{\pi D}^{2}}{4s}\;$$

where D is the bar diameter and s is the spacing. In the present study, a reinforcement arrangement consisting of #12 bars spaced at 150 mm in both directions, at both the top and bottom, was adopted, which corresponds to a thickness of approximately 0.75 mm for a single reinforcement layer. After defining the layered cross-section of the reinforced concrete slab, appropriate constitutive models are assigned to each layer. Reinforcement layers are modeled using a bilinear elasto-plastic material with isotropic hardening, incorporating the Bauschinger effect, based on the Menegotto–Pinto formulation [87] (OpenSees Steel02 uniaxial material model, implemented by Filippou et al. [88]). To simulate reinforcement fracture, the MinMax material wrapper is used in OpenSees, with the maximum steel strain set to ε_uk_ = 10%, corresponding to a yield strength of f_y_ = 500 MPa. The backbone of the steel stress–strain relationship, along with the associated input parameters (E_S_—initial elastic tangent; b—strain hardening ratio (ratio between post-yield tangent and initial elastic tangent); R0, cR1, cR2—additional parameters to control the smooth transition between the two lines of initial steel modulus of elasticity and the hardening modulus) is illustrated in Figure 5.

**Figure 6:**
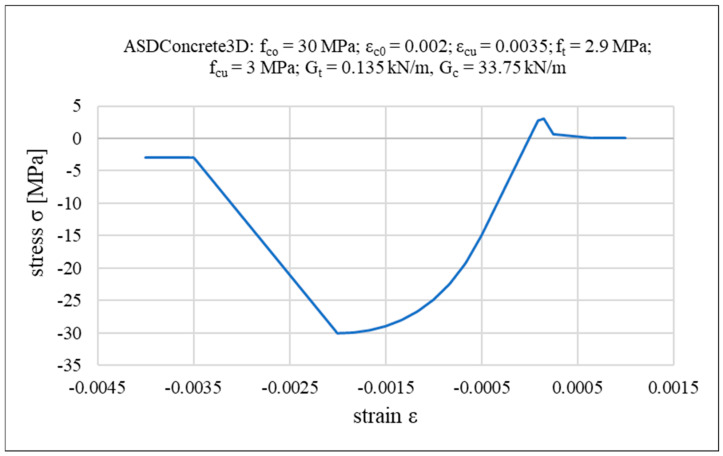
Backbone of the stress–strain relationships of concrete.

**Figure 7:**
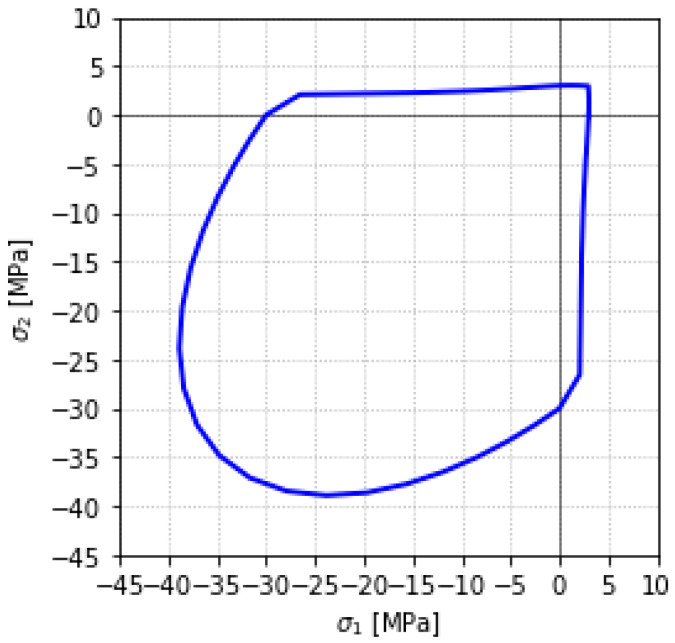
Damage surface for the biaxial compression/tension test.

For concrete, the ASDConcrete3D multidimensional constitutive material model is used [89]. A detailed description of this model can be found in [90,91,92]. It is based on the continuum damage theory, in which the stress tensor can be explicitly obtained from the total strain tensor, without internal iterations at the constitutive level. This makes it fast and robust, suitable for the simulation of large-scale structures. Plasticity is added in a simplified way, in order to have the overall effect of inelastic deformation but keeping the simplicity of continuum damage models. Depending on the required level of detail, the ASDConcrete3D material allows for different input parameter configurations. The available options include 1P, 4P, 6P, 9P, and fully user-defined settings. The 1P configuration requires only compressive strength as the input; the remaining parameters (e.g., tensile strength, ultimate strain, fracture energy) are automatically determined based on the Model Code 2010 recommendations [93,94]. In this study, a concrete compressive strength of f_co_ = 30 MPa is assumed using the 1P configuration. Note that if experimental data are available, more parameters can be specified explicitly. Accordingly, the analyzed system can be regarded as a potential structural configuration that could be realized in future practice. The backbone curve for the uniaxial stress–strain relationship and other specified concrete parameters (ε_c0_—concrete strain at maximum strength; ε_cu_—concrete strain at crushing strength; f_t_—tensile strength; f_cu_—concrete crushing strength; G_t_—tensile fracture energy; G_c_—compressive fracture energy) are shown in Figure 6, while Figure 7 illustrates the failure surface under biaxial compression/tension loading.

Due to the use of shell elements, the following modeling limitations are adopted:

- No slip is allowed between reinforcing bars and the surrounding concrete (perfect bond assumption);
- Bar pull-out failure in the connections is not considered;
- Out-of-plane shear failure of the slab is not taken into account;
- Failure of the columns is not taken into account;
- The influence of strain rate on structural strength is neglected.

The performance of nonlinear numerical analyzes, particularly when using an elasto-plastic concrete material model with damage, is partly dependent on the mesh density of the finite element discretization. However, it should be noted that the constitutive model ASDCONCRETE 3D implemented in this study incorporates an auto-regularization function. This function computes the specific fracture energy g_t,c_ (in tension and compression) per unit volume according to the following relation:

(2) $$g_{t,c}=G_{t,c}/l_{\mathrm{dis}}$$

where l_dis_ denotes the characteristic dimension of the finite element. Based on this relation, the final stress–strain response in tension and compression is obtained, ensuring that the total fracture energy G_t,c_ is preserved regardless of mesh refinement. In all analyzes presented in this paper, a finite element mesh size of 0.30 m × 0.30 m  is employed, providing an appropriate balance between computational accuracy and efficiency.

To overcome convergence problems, the Krylov–Newton [95] solution algorithm with adaptive time step is chosen. The convergence tolerance is initially set as 10^−6^ of the displacement increment norm.

## 3. Slab with Linear Concrete Material

**Figure 8:**
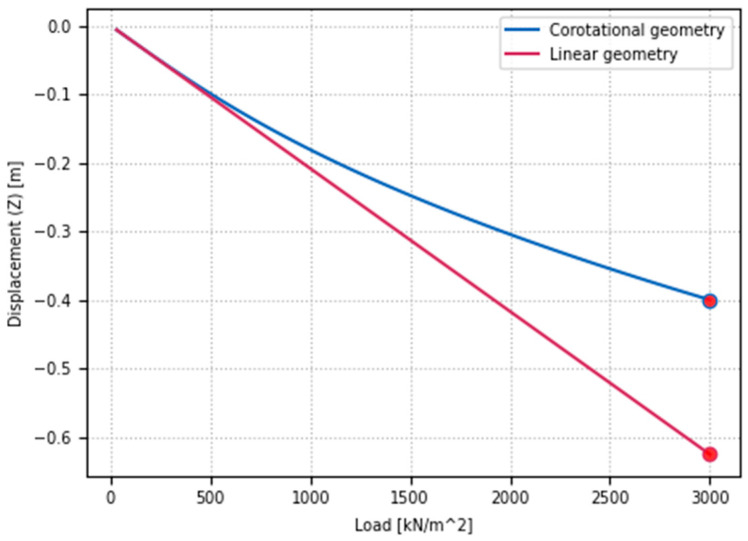
Comparison of geometric linearity and nonlinearity considerations in reinforced concrete slabs: mid-span deflection analysis.

This section presents the influence of geometric nonlinearity using the corotational formulation in comparison to a fully linear analysis. It should be noted that a multi-layer slab cross-section is also employed in this case; however, each layer is modeled as elastic, with a modulus of elasticity E = 30 GPa. For both analysis types, the mid-span deflection of the slab is monitored under increasing load, as illustrated in Figure 8.

**Figure 9:**
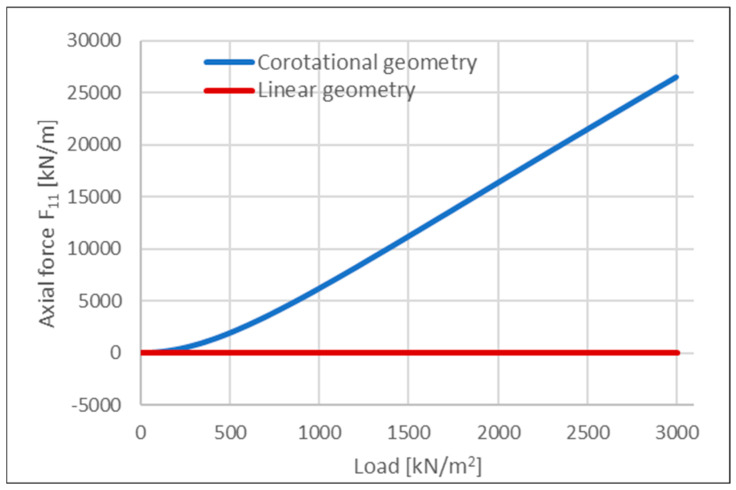
Comparative analysis of axial force evolution at the mid-span of a slab considering linear and corotational geometric formulation.

In the linear analysis, the final vertical deflection reached u_z,L_ = 0.63 m, whereas in the corotational formulation, it was reduced to u_z,C_ = 0.40 m. The decreased vertical displacement in the case of nonlinear geometry can be attributed to additional geometric stiffness resulting from the lateral constraints imposed on the slab and the development of axial tensile forces. A similar effect can be observed in beams where tension in elements contributes to the total element stiffness, e.g., [96]. The evolution of axial force in the selected direction at mid-span is shown in Figure 9.

**Figure 10:**
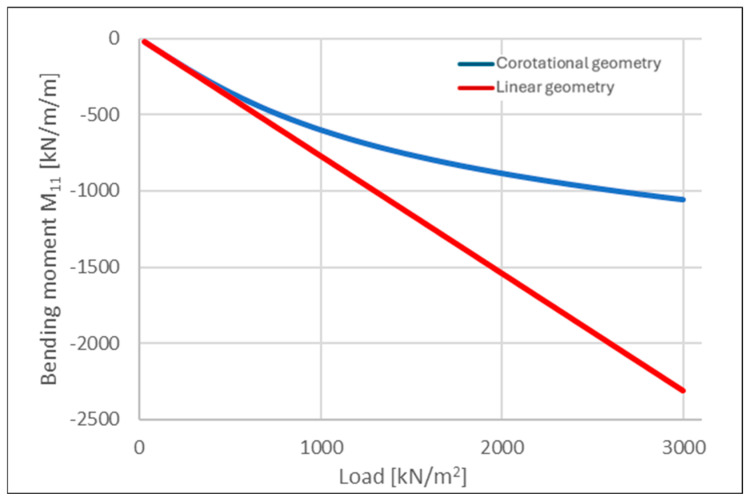
Comparative analysis of bending moment evolution at the mid-span of a slab, considering linear and corotational geometric formulation.

When analyzing the evolution of bending moments in a selected direction at mid-span (Figure 10; negative values indicate tension in the bottom fibers of the cross-section), the linear analysis resulted in a final bending moment approximately twice as large as that obtained from the corotational formulation. This difference is partially related to the reduced vertical deflections but also stems from the nature of the corotational theory, which solves the equilibrium equations iteratively with respect to the deformed configuration.

The lower bending moments observed in the geometrically nonlinear analysis can be attributed to the generation of horizontal support reactions. These reactions, when multiplied by the vertical displacements, reduce the net internal bending moment in comparison to the linear analysis, where such effects are not captured.

## 4. Slab with Nonlinear Concrete Material—Nonlinear Static Pushdown Analysis

**Figure 11:**
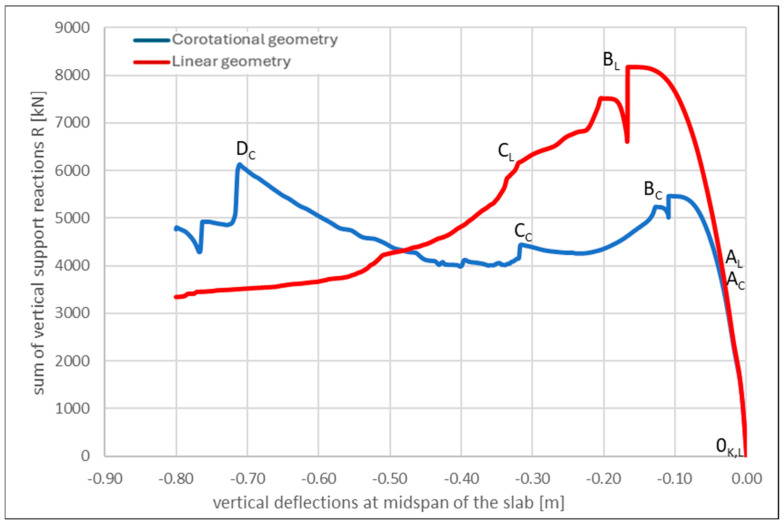
Comparison of pushdown curves for linear and corotational geometry with nonlinear concrete material model.

This section presents the results of the nonlinear static pushover analysis, in which the influence of linear and corotational kinematics on the load-carrying capacity and structural response (Figure 11) are compared. In both cases, a nonlinear damage-based concrete model is used, and the slab is subjected to incrementally increasing mid-span deflections up to u_z_ = 0.80 m using the displacement control method.

To illustrate the differences in structural behavior resulting from the assumed kinematics, additional notations are introduced along the load–deflection curves shown in Figure 11, corresponding to specific characteristic points. As can be observed, both the linear and corotational analyzes exhibit curves with similar shapes in the intervals from 0_C,L_ to A_L_, B_L_ and from 0_C,L_ to A_C_, B_C_, respectively, although the associated load levels differ significantly. Reaching points A_L_ and A_C_ in both analyzes corresponds to the onset of yielding in the top reinforcement along the slab edges |AB| and |CD|, starting from the midpoint of each edge and propagating toward the column region. For both points, this occurs at vertical deflections of u_z,L,C_ = 0.03 m, with the corresponding total applied load for point A_L_ being 3143 kN, which translates to a uniformly distributed load of 3143 kN/(9.00 m × 6.00 m) = 63 kN/m^2^, and for point A_C_, 3074 kN/(9.00 m × 6.00 m) = 57 kN/m^2^. Up to this stage, both the linear and corotational analyzes provide similar structural responses.

Points B_L_ and B_C_ are characterized by continued yielding of the reinforcement and the onset of compression in the lower concrete layers, occurring both along the slab edges and at mid-span. These points represent the maximum flexural capacity of the structure, beyond which catenary action becomes active, as evidenced by a distinctive jump in the load–bearing curves. For point B_L_, this transition occurs at a total applied load of 8178 kN, corresponding to a uniformly distributed load of 8178 kN/(9.00 m × 6.00 m) = 151 kN/m^2^. For point B_C_, the total load is 5455 kN, which equates to 5455 kN/(9.00 m × 6.00 m) = 101 kN/m^2^. At this stage, the flexural resistance along the longer edges of the slab is primarily sustained by the top reinforcement and the residual tensile capacity of the concrete fibers. It is worth noting that a similar shape of the pushdown curve under corotational nonlinearity condition is also reported by Yang et al. [97].

The analysis is terminated after a sudden drop in load-carrying capacity at point D_C_, where the top reinforcement along the shorter slab edges |AC| and |BD| fractured. Beyond this point, the structural capacity is provided solely by the bottom reinforcement and the remaining uncrushed concrete. The response state observed at point D_C_ (u_z,C_ = 0.72 m, q = 112 kN/m^2^) is referred to as a pure catenary mechanism.

**Figure 12:**
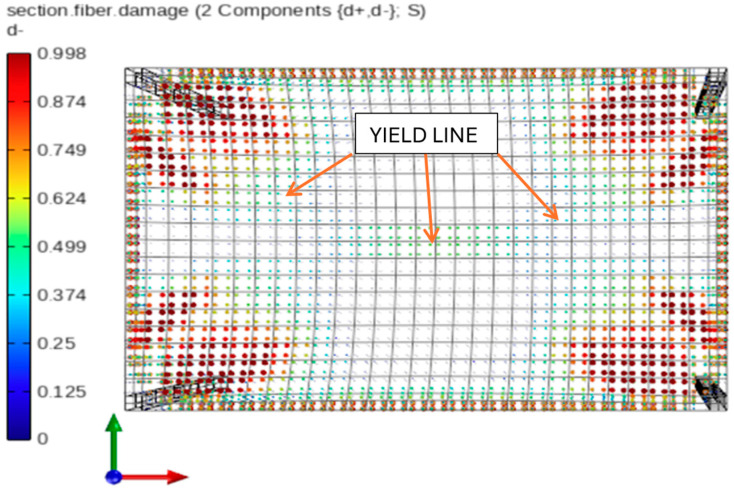
Compression fiber damage distribution in concrete under corotational geometry and nonlinear material model.

**Figure 13:**
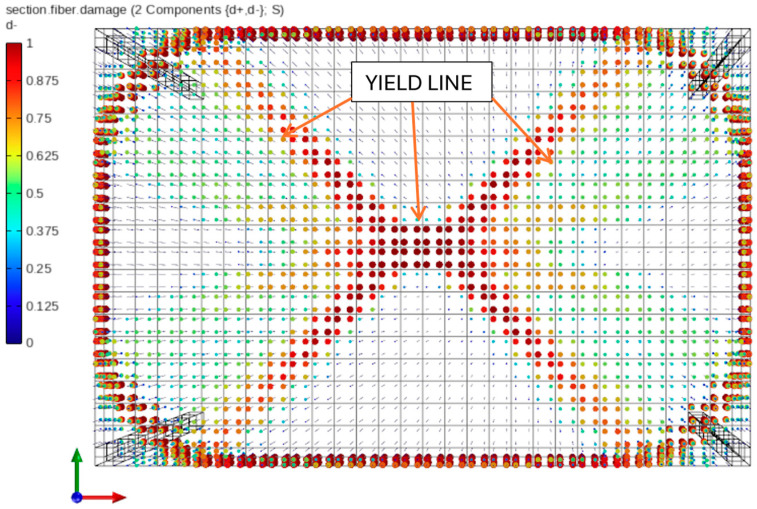
Compression fiber damage distribution in concrete under linear geometry and nonlinear material model.

In STKO, post-processing maps can be generated for each analysis step and each layer to visualize equivalent plastic strains, tensile damage and cracking levels, principal stresses, and other quantities. For simulations focused on damage propagation, a particularly useful feature is the ability to extract results in terms of compressive damage (d^−^) and tensile damage (d^+^) components. These scalar damage factors range from 0 to 1, where 1 indicates complete material failure. According to the constitutive behavior of the ASDConcrete3D model (Figure 6), the d^−^ value of 1 corresponds to the attainment of residual compressive strength f_cu_ and ultimate compressive strain ε_cu_, while the material is considered undamaged when the compressive strain remains below ε_c0_. To further evaluate the influence of geometric nonlinearity, Figure 12 and Figure 13 present concrete damage maps in compression for a deflection level of u_z,C,L_ = 0.80 m. A greater extent of concrete damage is observed in the linear analysis, suggesting a more advanced degradation state compared with the corotational case.

The main difference lies in the extent of crushing observed in the compressed concrete fibers at mid-span. In the linear geometry case, full crushing occurred in this region, whereas in the corotational case, the same fibers reached only half of the damage threshold (d^−^ = 0.50), corresponding to a plastic compressive strain of ε_c,plastic_ = 0.00275. It is also worth noting that in both cases, the formation of characteristic yield lines is observed, as previously illustrated in Figure 1.

**Figure 14:**
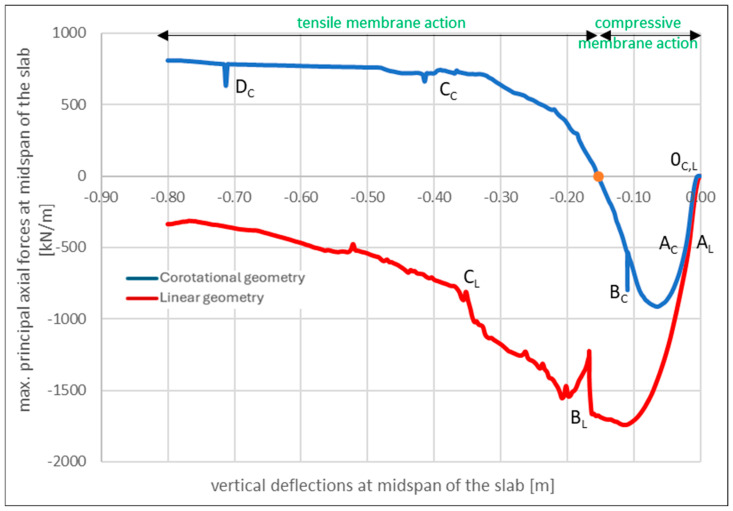
Development of principal axial forces at the mid-span of the slab.

When analyzing the curves presented in Figure 11, it can be observed that neglecting geometric nonlinearity results in a higher apparent slab capacity. This outcome theoretically undermines the effectiveness of the structural resistance mechanisms described earlier. The explanation for this behavior lies in the analysis of axial force development in the slab (Figure 14), which is most clearly interpreted using max. principal internal forces, representing the extreme values of internal force components within the shell elements.

Figure 14 shows the absence of axial tensile forces in the analysis with linear geometry. This explains both the initially higher load resistance (primarily carried by the concrete) and the more severe damage observed at the final stage of the analysis, where the concrete fibers at mid-span experienced progressive crushing prior to the fracture of the top reinforcement. Notably, no distinct transition to the D_L_ phase is observed.

This behavior is in stark contrast to the corotational analysis, in which a characteristic sign change in axial forces occurs at a deflection of approximately u_z,C_ = 0.15 m, corresponding closely to the effective depth of the slab. This transition confirms the activation of the membrane resistance mechanism described in the literature (see, e.g., [66]). It marks the shift from CMA to TMA, which ultimately leads to an increase in load-carrying capacity once deflections exceed u_z,C,L_ = 0.48 m (see Figure 11).

## 5. Discussion

In the case of progressive collapse, the structure experiences significant deformations, which leads to an increasing divergence between the results of linear and nonlinear geometric analyzes. To address this issue, a nonlinear static pushdown analysis is carried out. The key result of this analysis is the load–displacement curve that describes the relationship between the total vertical reaction and the corresponding vertical deflection. This study expands the scope of nonlinear static analysis by presenting results that consider material nonlinearity along with both linear and nonlinear geometric assumptions.

**Figure 15:**
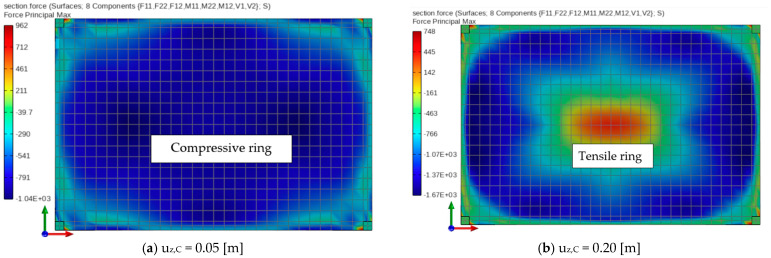

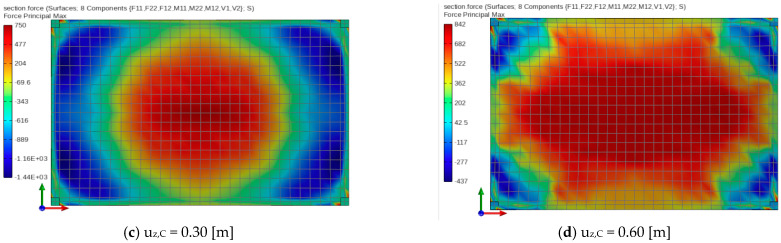
Phases of development of the compressive and tensile ring for the full nonlinear analysis depending on the mid-span deflections of the slab.

It is important to emphasize that the present work demonstrates that a membrane resistance mechanism can only be captured numerically when both material and geometric nonlinearities are simultaneously accounted for. This mechanism involves the formation of a central tensile ring and an adjacent compressive ring along the slab edges. In the example with nonlinear geometry only (see Figure 9), the slab mid-span is subjected exclusively to tensile forces. Conversely, in the case involving material nonlinearity and linear geometry, only compressive behavior is observed (see Figure 14). These findings indicate that only the combination of both nonlinear effects enables the accurate development and propagation of tensile and compressive rings under progressive collapse conditions. This behavior is illustrated in Figure 15, which presents principal axial force maps at various stages of the analysis.

A particularly significant aspect of this analysis is the observed transition from TMA to CMA, which occurred once the vertical deflection reached the effective depth of the slab. This behavior confirms assumptions made in earlier studies reported in the literature and is not captured in any of the linear analyzes.

## 6. Conclusions

The behavior of a flat-slab structure is investigated by performing four static analyzes, tracking the evolution of deflections, bending moments, and axial forces. By employing the ASDConcrete3D nonlinear concrete material model with damage and the ASDShellQ4 multi-layer shell element, it is possible to capture the progressive crushing of concrete in the compressive zones and yielding of reinforcement in the tensile layers. This highlights the level of accuracy and control achievable in advanced nonlinear simulations of this type.

In the linear analysis (Section 3), the load increment did not induce axial forces in the slab, which resulted in greater bending moments and deflections compared with the analysis employing the corotational formulation. In the full nonlinear model (Section 4), a progressive failure mechanism is demonstrated. Using the displacement control method, characteristic points associated with the formation of plastic hinges are identified on the pushdown curve. It is noteworthy that, in the full nonlinear model, a smaller extent of the damaged slab area was observed; in particular, at mid-span, only approximately half of the plastic deformations were reached. In contrast at the same place, in the model with linear geometry, complete crushing of the compressed concrete fibers occurred. This finding indicates the effective action of the membrane resistance mechanism in mitigating the effects of progressive collapse.

It is demonstrated that only a fully nonlinear analysis is capable of realistically capturing structural behavior under large deformations, with the transition from a compressive to a tensile membrane resistance mechanism identified as a particularly critical stage. This finding may provide an important reference for future revisions of design codes addressing progressive collapse where, for example, the current provisions of the GSA guidelines [5,6] still permit the use of linear analyzes. Moreover, the application of fully nonlinear global analysis, consistent with structural design practice, inherently accounts for geometric nonlinearity and second-order effects, making additional stability checks unnecessary. In this way, simplified approaches such as separate stability verifications or approximate second-order analyzes are no longer required.

The results presented herein may also serve as a basis for designing future experimental studies on selected structural subassemblies or as reference data for validating more complex numerical models of full-scale structural systems.

It should be emphasized that the nonlinear static pushdown analysis employed in this study provides a means of estimating the maximum load-carrying capacity of the structure. In the context of progressive collapse, it is particularly useful for evaluating alternative load paths, which is consistent with strategies aimed at limiting the extent of local failure, as outlined in design codes such as Eurocode [1].

Moreover, since progressive collapse may also be triggered by sudden column loss or similar localized damage, future studies will focus on nonlinear dynamic analyzes, incorporating inertia effects, damping, and structural vibrations.

## Data Availability

The raw data supporting the conclusions of this article will be made available by the authors on request.

## Abbreviations

The following abbreviations are used in this manuscript:

STKO	Scientific Toolkit for OpenSees
OpenSees	Open System for Earthquake Engineering Simulation
FEM	Finite Element Method
CAA	Compressive arch action
CTA	Catenary tension action
TMA	Tension membrane action
CMA	Compression membrane action
